# Advances in Organic Rheology-Modifiers (Chemical Admixtures) and Their Effects on the Rheological Properties of Cement-Based Materials

**DOI:** 10.3390/ma15248730

**Published:** 2022-12-07

**Authors:** Qianqian Zhang, Jian Chen, Jiang Zhu, Yong Yang, Dongliang Zhou, Tao Wang, Xin Shu, Min Qiao

**Affiliations:** 1 State Key Laboratory of High Performance Civil Engineering Materials, Jiangsu Sobute New Materials Co., Ltd., Nanjing 211103, China; 2 Shandong Provincial Key Laboratory of Preparation and Measurement of Building Materials, University of Jinan, Jinan 250022, China

**Keywords:** superplasticizer, viscosity-modifying admixture (VMA), rheology, cement-based material

## Abstract

Organic rheology modifiers, especially superplasticizers and viscosity-modifying admixtures (VMAs), have become key components for the workability optimization of modern concrete. The development of these admixtures is crucial to the further performance improvement of modern concrete under different casting and service conditions. Many of the former reviews have summarized research advances in respect of these admixtures from chemical and material perspectives, focusing on the effects of structure and the performance. In this paper, from a rheological perspective, an overview is provided of the microscale behavior of polycarboxylate (PCE) superplasticizers and VMAs (e.g., adsorption, conformation, and bridging) in terms of the evolution of the microstructure of the paste, the effect of chemical structure on the yield stress, the apparent viscosity and thixotropy of cement-based materials, and the structure design of these admixtures. Most importantly, in addition to a general discussion with assumptions (monolayer adsorption of a “flat” conformation, with each molecule on a single particle; statistical polymer composition), special conditions (e.g., preferential adsorption, depletion effects, hydration modification effects, and the polydispersity of the polymer composition) are discussed. Newly developed admixtures, realized through regulation of the microscale behavior, and by the modification of adsorption, topological structure, and molecular frame, are introduced.

## 1. Introduction

With the rapid growth of the global population, Earth’s natural resources and environment are experiencing unprecedented demand. To meet the growing residential and infrastructure demand in a densely populated world, construction materials exhibiting high performance, long life, and versatile functionality are becoming the future trend. As concrete is the most widely used construction material, global demand for concrete has remained high in recent years, with consumption even growing in emerging markets. Further improvements and refinements in performance, durability, and functionality are the main goals for industry and researchers.

To ensure smooth processes in casting and construction, and to guarantee the final quality of cement concrete projects, optimization and control of concrete workability are of great significance. Nowadays, various types of concrete have been developed, with distinctly different workability requirements. For instance, ready-mix concrete, which is prepared in a mixing station, should be transported to the construction location and is generally casted by pumping. Thus, long-term slump retention until final casting and low shear-stress during pumping are required. For self-consolidating concrete, high flowability without bleeding and segregation is crucial. For ultra-high-strength concrete with a low w/b (e.g., 0.2), rapid structural-build up might occur and the fluidity after standing for only minutes can be lost until complete remixing is performed [1,2]. For pre-cast concrete, the workability should match the casting and curing procedure, especially before and after molding. In addition, concrete should retain high stability and proper flowability before and during molding, otherwise the quality and shape of the prefabricated components cannot be ensured. Moreover, 3D printing [3,4] techniques, which can reduce the labor cost and are especially suitable for concrete with a unique appearance, have been introduced to cement-based materials in recent years. The material before casting should have low viscosity under high shearing, while rapid flowability loss is required to hold the shape. In principle, concrete should remain uniform at all stages. The flowability at different stages should be adjusted depending on the requirements.

Since the invention of chemical admixtures, workability could be ensured at a low water to binder ratio (w/b ratio), while incorporation of high-dosage supplementary cementitious materials has become possible [5]. Chemical admixtures, especially organic rheology modifiers, have become key components for the workability optimization of modern concrete with high performance. Superplasticizers, which are polymer dispersants, can reduce the w/b ratio to 0.14, indicating concrete strength of over 150 MPa. Natural and synthetic organic VMAs are also commonly used to improve concrete stability. The development of these admixtures has attracted tremendous attention. Previous reports have reviewed the development of superplasticizers [6,7,8] and VMAs during the past decades [9]. Some other reports, focusing on the adsorption of superplasticizers [10,11], have also been published. However, most of these reports have focused on the effect of the chemical structure and the performance of admixtures. From a rheological point of view, the development of organic rheology modifiers, especially in terms of their structure-related interaction with cement-based materials and their effects on the rheological behavior of cement-based materials, has not yet been extensively reviewed. Herein, in this paper we briefly review the fundamental mechanisms of the rheology (yield stress, apparent viscosity, and thixotropy) of cement-based materials in the presence of rheology modifiers. The working mechanisms, design, and improvement of superplasticizers and VMAs are also summarized.

## 2. Rheological Properties of Cement-Based Materials

**Figure 1:**
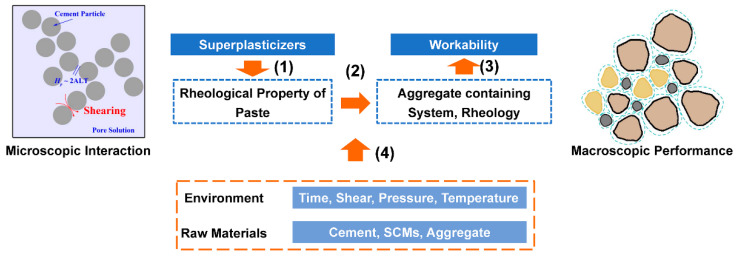
The four aspects (orange arrows represent the relationships) to elucidate the effects of chemical admixtures on concrete workability.

Concrete is a complex system, with components ranging from ~10^−2^ m (e.g., gravel) to ~10^−9^ m (e.g., molecules of chemical admixtures), and continuous hydration over months and years. To elucidate the effect of organic chemical admixtures on concrete workability, the subject is divided into the following four aspects (Figure 1): (1) The effect of organic chemical admixtures on the rheological properties of paste, which involves time-dependent physical and chemical interactions; (2) build-up of concrete rheology from paste rheology, by incorporating aggregate-related calculations and considering the paste to be a uniform material with certain rheological properties; (3) the relationship between concrete rheology and concrete workability; (4) the impact on rheology and workability considering different environmental factors, operation conditions, and binder systems. Generally, when supplementary cementitious materials, manufactured sand with micro-fine particles and clay contaminants [12] are incorporated, workability problems (e.g., low initial slump, bleeding and segregation, rapid slump loss, and flash set) can always be observed. The unfavorable performance is identified to be a result of the incompatibility of chemical admixtures with the raw materials or environmental conditions [13,14,15].

The effects of organic chemical admixtures are primarily exerted at the microscale (10^−9^~10^−5^ m). Therefore, the first focus of this review is the effect of chemical admixtures on the time-dependent shearing-response characteristics (time-dependent yield stress, apparent viscosity, and thixotropy) of paste. These characteristics are firstly explained.

Generally, yield stress, which depends on the energy required to break the interparticle network [16,17], is the major factor determining the flowability of cement paste. Flowability of cement paste is usually measured by the fluidity value, which is the diameter of the paste. Therefore, the fluidity is a direct characterization of the yield stress of cement paste [18,19,20]. On a physical basis, yield stress represents the force required to make the paste deform. The paste stops spreading only when gravity cannot deform the paste any more. The resistance against the deformation is governed by the yield stress and surface tension of the paste cake. On a deeper level, yield stress is determined by particle interactions (colloidal forces, e.g., attractive van der Waals interaction, static charge interaction). These interactions depend on the strength of the particle interaction, which clearly depends on the distance between particles (the yield stress model—YoDEL, developed by Flatt and Bowen [21,22]):

$$\tau_{0}≅m\frac{A_{0}a^{*}}{d^{2}H^{2}}\times \frac{\varphi^{2}\left(\varphi -\varphi_{perc}\right)}{\varphi_{m}\left(\varphi_{m}-\varphi \right)}$$

where *A*_0_ is the Hamaker constant; a* is the particle shape parameter [17]; *d* is the average particle diameter; *H* is the surface-to-surface separation distance at contact points; *ϕ*, *ϕ_perc_*, and *ϕ_m_* are the volume fraction, percolation volume fraction, and volume fraction of maximum packing density, respectively; m is the pre-factor.

The difficulty of any operation or handling of the paste is determined by the viscosity (apparent viscosity at a certain shear rate). The apparent viscosity of cement paste is the energy required to destroy the colloidal interaction network between cement particles (yield stress) and overcome the hydrodynamic force during shearing and particle inertia (residual viscosity) [16,17]. The hydrodynamic force is the resistance induced by the shearing of the viscous continuous phase toward flow and particle motion. It depends on the local shear intensity between particles and the viscosity of the continuous phase. At a consistent shear rate, the shear intensity is proposed to be reversely correlated with the distance between cement particles [16,23]. At a low w/b ratio and a high shear stress, shear thickening can always be observed due to a non-linearly growing inertial force [17,24] or enhanced particle friction [20]. As reported by [16], the deflocculation process of the cement paste can reduce the apparent viscosity due to the increased distance between particles.

Generally, three stages of shear viscosity can be identified, and within each stage, the dominate interaction is different [16,17]. At a low shear rate, the particle flocculation can be destroyed and a rapid decrease in shear stress and shear viscosity can be observed. Colloidal forces dominate the interaction during this stage. At a higher shear rate, the hydrodynamic forces become significant and the shear viscosity reaches a lower plateau; this is called pseudo-Newtonian viscosity. At an even higher shear rate, inertial force ($\propto {\dot{\gamma }}^{2}$) dominates, and shear thickening might be observed. In the following discussion, we focus on the second stage, due to the similar range of shear rates in general applications (10^0^~10^1^ s^−1^). Additionally, the influence of the particle network can be neglected due to the same flow spread (yield stress).

In addition, it should be noted that during the mixing process of a paste, hydration simultaneously occurs. Particles dissolve, new hydration products form with water consumed, and particles are bonded and connected. The interparticle network becomes stronger, which induces loss of fluidity. The strength of the network comes from the colloidal forces and the particle bonding via hydration products (e.g., C-S-H) [25,26]. Indeed, the colloidal interaction can be interrupted by shearing, but recovers if shearing is stopped. The particle bonding via hydration products will be destroyed over a certain strain (10^−9^ m). A critical shear stress (static yield stress) would be required to overcome the particle network from a standing state to make the paste deform. Models have been established for the increase in static yield stress [27,28]. Within an early time period (dominated by early hydration of the aluminate phase), the increase in static yield stress primarily depends on colloidal interactions, while bridging interactions slowly enhance the network and make the static yield stress increase with time.

In the presence of organic chemical admixtures, the development of particle networks becomes much more complicated, since the admixtures can bind metal ions in solution, adsorb on clinker and hydration products, and affect both the hydration process and the interactions between particles.

## 3. Superplasticizers

In a superplasticized paste, superplasticizers adsorb on the surface of cement particles and early hydration products, and weaken the attractive van der Waals forces via static charge repulsion and steric hindrance. In this section we focus on polycarboxylate-type superplasticizers, which exert their effects via steric hindrance. As the most common superplasticizer, polycarboxylate (PCE) superplasticizer is a type of comb-like polymer with an ionic backbone and polyethylene glycol (PEG) side chains. Once adsorbed, steric hindrance from PEG chains will prevent particles from forming flocs.

**Figure 2:**
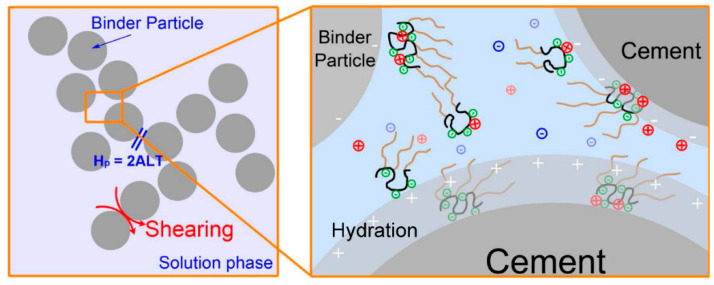
Microscale view of cement paste and different forms of PCE molecules in cement paste.

Generally, PCE in a paste is present in three forms (Figure 2): (1) an initial part of the PCE—this form is consumed by hydration to form organo-mineral phases [29,30]. It does not contribute to particle dispersing, but the consumption is associated with so-called “cement compatibility”, since cements containing different amounts of aluminate phase and gypsum differ from each other in early hydration; (2) PCE that is adsorbed on the particle surface to provide steric hindrance; (3) PCE that remains in the solution phase—this form affects the solution viscosity and contributes to the particle interaction as reported by [31].

The particle separation distance is correlated with the adsorption layer thickness [16,32]:

$$\frac{1}{H^{2}}=\frac{\theta^{2}}{H_{p}^{2}}+8\frac{\theta \left(1-\theta \right)}{{\left(H_{p}+H_{0}\right)}^{2}}+\frac{{\left(1-\theta \right)}^{2}}{H_{0}^{2}}$$

where *H*_0_ represents the average particle distance without any additive (e.g., PCE), while *H_p_* is the particle separation distance at a full surface coverage of PCE.

It should be noted that forms (2) and (3) also affect the dissolution and nucleation of solid particles, and thus the amount, size, and morphology of hydration products at varied times, which will further affect the packing and particle network. All these effects on paste rheology depend on the adsorption affinity and PCE conformation.

### 3.1. PCE Conformation and Adsorption

#### 3.1.1. Solution and Adsorption Conformation

The solution conformation of PCE superplasticizers determines the solution viscosity and adsorption process. Solution conformation of PCEs as well as the molecular size can be characterized by light scattering and small-angle neutron scattering [33]. For PCE molecules in alkaline solution, the conformation primarily depends on the balance between intramolecular (e.g., charge repulsion from deionized carboxylic groups) and intermolecular interactions (e.g., binding with counter-ions) [34,35,36,37] and references therein]. Charge repulsion from the deionized carboxylic groups leads to stretching of the polymer chain, while charge-screening effects via counter-ions (e.g., Ca(II)) induces coiling. Different forms of Ca(II) binding will further result in different solution behaviors. For a single PCE molecule and Ca(II) binding with a single carboxylic group, the effect of Ca(II) is similar to that of strong counterions. For a single PCE molecule and Ca(II) binding with multi-carboxylic groups (PCE with a long backbone and low grafting density), a collapsed backbone with an especially small size will be observed. For Ca(II) binding between multi-PCE molecules, aggregates will form.

Due to the complicated environment in cement paste, direct measurement of the adsorption conformation of PCE is difficult. Recently, reports regarding computational modeling of the conformation of PCE superplasticizers have been published (for details, please refer to [38,39], which will not be discussed here). The measurement of adsorption layer thickness has been reported by [40,41,42,43]. Atomic force microscopy and dynamic light scattering, in which the size change before and after PCE adsorption can be calculated, are also effective approaches.

**Figure 3:**
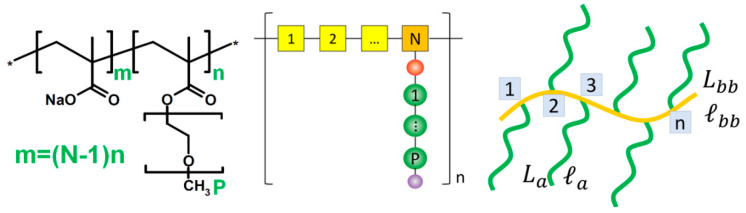
Model structure of comb-like PCE (polyethylene glycol monomethyl ether grafting on poly(methacrylic acid) backbone). Chemical structure (**left**); schematic representation of the comb−like polymer for scaling model (**middle**); schematic representation of a comb-like structure (**right**).

However, for rapid evaluation, scaling models are still required. To aid discussion of the conformation, a PCE model is briefly introduced (Figure 3). This model was initially proposed by Gay and Raphael [44]. Therein, the polymer contains *n* segments, each with one side chain. Each side chain contains *P* repeat units. Each segment contains *N* backbone monomers.

As reported by Flatt [43], for PCE molecules with a flexible backbone worm (FBW) conformation, the radius (corresponding to adsorption layer thickness at full surface coverage) of a blob can be determined by the chain parameters:

$$R_{\mathrm{AC}}={\left(2\sqrt{2}\left(1-2\chi \right)\frac{a_{p}}{a_{N}}\right)}^{1/5}a_{p}P^{7/10}N^{-1/10}$$

while one polymer molecule takes up a certain part of occupied area *S* [43] for single-layer adsorption:

$$S=\frac{\pi }{\sqrt{2}}a_{N}a_{p}{\left(2\sqrt{2}\left(1-2\chi \right)\frac{a_{p}}{a_{N}}\right)}^{2/5}P^{9/10}N^{3/10}$$

where *a_N_* and *a_p_* stand for the size of the repeat unit of the backbone and side chain, respectively; χ is the Flory–Huggins parameter. For the PCE here, *a_N_* and *a_p_* are 0.25 nm (the size of an acrylate or a methacrylate unit in the backbone) and 0.36 nm (the size of an ethylene glycol unit), respectively. χ = 0.37 for polyethylene in an aqueous solution at 25 °C.

By taking the excluded volume of chain units into account, Wang et al. developed another model by which to calculate the solution conformation and adsorption conformation [45,46,47,48]. The mean square end-to-end distance of the “train-like” adsorbed backbone and the surface occupation area of each PCE molecule can be evaluated:

$$R^{2}{}_{bb,2D}=\{\begin{array}{c} 2l_{bb,2D}^{2}\left[\frac{L_{bb}}{l_{bb,2D}}+exp\left(-\frac{L_{bb}}{l_{bb,2D}}\right)-1\right],L_{bb}/l_{bb,2D}\leq 2.1491\\ 0.8035l_{bb,2D}^{2}{\left(L_{bb}/l_{bb,2D}\right)}^{\frac{3}{2}},L_{bb}/l_{bb,2D}>2.1491 \end{array}\;S_{A}≅\pi R^{2}{}_{bb,2D}/4$$

Using a surface-tethered chain model, the local adsorption layer thickness can be evaluated to be:

$$H_{ads}≅\{\begin{array}{c} 2R_{g,PEG},\sigma \leq {\left(2R_{g,PEG}\right)}^{-2}\left(\mathrm{Mushroom}\right)\\ 0.7L_{a}{\left(\sigma l_{a}^{2}\right)}^{1/3},\sigma >{\left(2R_{g,PEG}\right)}^{-2}\left(\mathrm{Brush}-\mathrm{like}\right) \end{array}$$

where *L_bb_* and *L_a_* are the counter length of the backbone and the grafting polymer arm, respectively; *l_bb_* and *l_a_* are the persistent length of the backbone and grafting polymer arm, respectively; *σ* is the local side chain density; *R_g_* is the radius of gyration.

**Figure 4:**
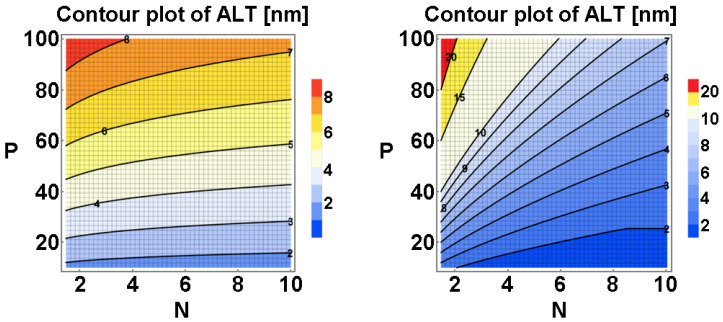
Contour plots of the adsorption layer thickness (ALT) at high surface coverage; results are insensitive to the numbers of side chains (n): the Flatt model (**left**) and the surface-tethered chain model (**right**); data taken from [47].

The two models work well in most cases (Figure 4). The results are similar but not the same. By taking backbone stiffness into account, the end-to-end distance of the backbone will be much larger than in Flatt’s model. Additionally, as mentioned in [47], some assumptions are required for the models: (1) the side chains are assumed to be freely jointed to the backbone; (2) the polymer backbone “flatly” attaches to the particle surface to adopt a “train-like” conformation. Some other conditions (e.g., multi-layer adsorption and other adsorption conformations) might also be observed in application (as per the following discussion in Section 3.3). The models might not be applicable.

The maximal adsorption amount of polymer (A_s_) on powder can be evaluated to be SSA/Sm_PCE_ (SSA, specific surface area; m_PCE_, mass of a single molecule). A comb-like polymer with a long side chain and high grafting density will result in high values of A_s_ and H_p_.

#### 3.1.2. Adsorption Behavior

From a thermodynamics perspective, the adsorption of a PCE molecule on a solid particle is affected by the following interactions [49,50]: (1) attractive surface interactions (e.g., static charge attraction, chemical binding); (2) entropy gain from the release of ions (and water molecules) into the bulk solution; (3) loss of polymer conformational entropy owing to the surface exclusion effect; (4) steric hindrance and charge repulsion from the surface-adsorbed molecules. (1) and (2) promote adsorption, while (3) and (4) discourage adsorption. Generally, negatively charged PCE can adsorb on positively charged particle surfaces through static charge attraction, while on negatively charged particles, Ca(II) binding is crucial. For PCE with long side chains, the entropy gain is also significant in improving the adsorption.

The Gibbs adsorption free energy can be expressed as follows [48]:

$$\frac{\Delta G^{{}^{\circ}}}{RT}=-nz\left(N-1\right)\alpha +\frac{\alpha_{N}nN}{\lambda }+n\beta -n\left(N-1\right)\gamma$$

where α (dimensionless) represents the interaction of single adsorption group with the surface; *n* is the amount of the smallest repeat unit of the PCE backbone, which contains *N* backbone units and a single grafting side chain; *z* is the charge number of the adsorption group; *N* is the number of backbone units in each repeat unit; *nz*(*N* − 1) is the total charge amount of a single PCE molecule. *β* (dimensionless) represents the entropy loss of each side chain; *λ* is the deflection segment length determined by Odijk (refer to references in [48]); *γ* (dimensionless; between approximately 0 and 3; refer to references in [48]) represents the entropy gain of each water molecule or counterion into the solution phase.

Using this model, the adsorption affinity between different polymers can be compared. Adsorption can be promoted with an increased acid-to-ether/ester ratio (*N* increase) and chain stiffness (large *λ*, as already verified by experiments in [51]), and inhibited by increasing the side-chain length (β increase). For fixed *N* and *P*, an *n* increase (backbone length, or molecular weight) can also promote adsorption if ΔG° < 0.

As for models characterizing adsorption behavior, the Langmuir model and some other adsorption models are commonly applied. However, findings by Wang [47,48] indicate that the surface coverage cannot reach as high as that suggested by the Langmuir model, due to the excluded volume between polymer chains. In application, due to hydration, the adsorption behavior becomes even more complicated. Still, in the analysis of yield stress, surface coverage values calculated from adsorption and maximum adsorption amounts are effective.

### 3.2. Effect of Chemical Structure on Yield Stress and Apparent Viscosity of Cement Paste

Theoretically, all the parameters that affect the yield stress of powder dispersion (e.g., cement paste) affect the performance of polymer dispersants (e.g., PCE superplasticizer). For a certain powder dispersion based on the following conditions, the yield stress of the powder dispersion depends on the separation distance between particles and the true particle packing state: (a) particles with identical surface composition; (b) fixed water-to-powder ratio and particle morphology, indicating fixed theoretical volume fraction and maximum packing state; (c) mono-layer adsorption of polymer dispersant (e.g., Langmuir adsorption) on the particle surface. The average particle separation distance is determined by the surface coverage and *H_p_* [16,32]. Based on a flat adsorption model (polymer backbone flatly attached to the surface without loops), the *H_p_* of a comb-like polymer can be calculated to be twice that of the adsorption layer thickness at full surface coverage [32]. More specifically, since PEG side chains primarily provide steric hindrance, the adsorption layer can be considered to be PEG chains locally attached to the particle surface. Based on the surface-tethered chain model [47], at full surface coverage, *H_p_* should be determined by the length and density of the side chain “anchored” to the surface of particles.

**Figure 5:**
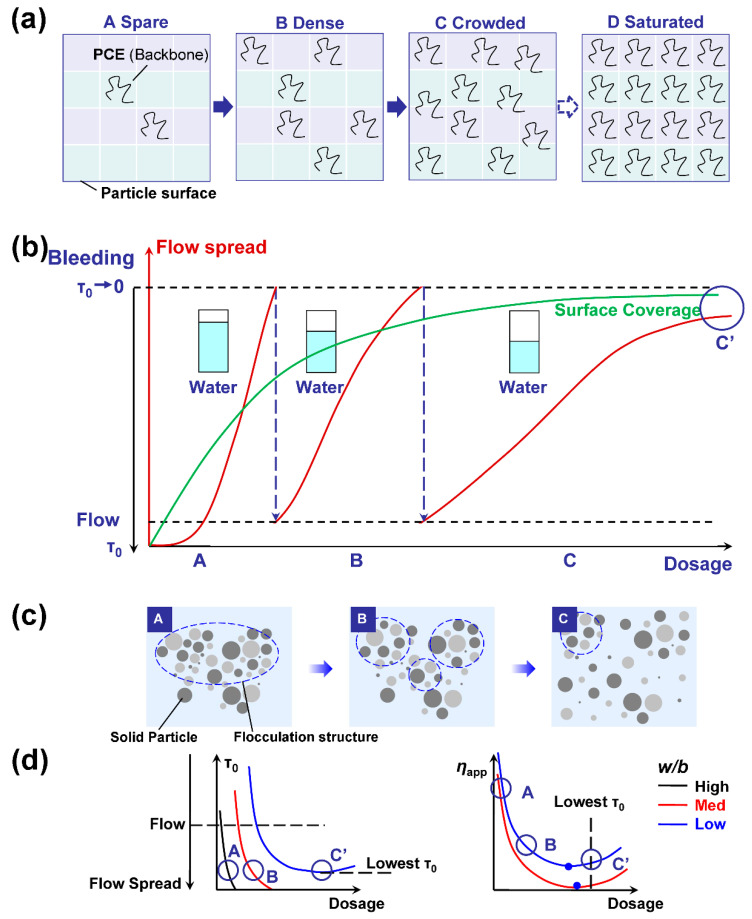
Effect of PCE dosage on the surface coverage (**a**), flow behavior (**b**), flocculation structure (**c**), yield stress (left in (**d**)) and apparent viscosity (right in (**d**)) of cement paste at varied w/b. A, B, C, and C′ in (**b**–**d**) indicate the same PCE dosages. The surface coverage of A, B, and C in (**b**–**d**) is in the range of A, B, and C in (**a**). C′ stands for full surface coverage (by experimental testing). (**a**) Each PCE backbone occupies a small grid on the particle surface without overlap (monolayer adsorption). From A to D, more and more PCE molecules adsorb on the surface. However, the PCE molecules are randomly distributed but not arranged in order on the surface in C. The surface saturation state D should be difficult to reach. (**b**) Flow spread of cement paste against PCE dosage at varied w/b. At C′, the flow spread reaches the maximal value. (**c**) The flocculation degree becomes lower and lower with the increase in PCE dosage (surface coverage). Due to preferential adsorption of PCE on different mineral phases, low surface coverage on a negatively charged surface should be a common condition, especially for PCE of extremely low adsorption affinity. The flocculation degree at C′ can still be reduced by using another type of PCE. (**d**) At least two stages evolution of yield stress (decrease to plateau) against dosage can be observed within the dosage range of common application. Few reports have demonstrated the increase in yield stress at extremely high PCE dosages for common PCE samples, due to depletion [59] or bridging [60,61]. Three stages of evolution of apparent viscosity can be observed (decrease, plateau, and increase).

**Figure 6:**
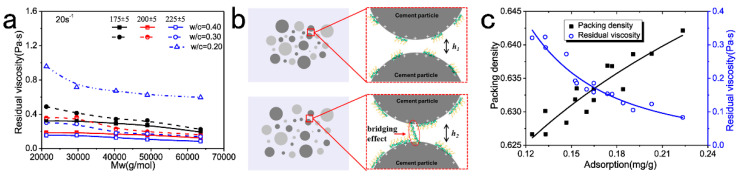
Residual viscosity of cement paste at 20 s^−1^ (**a**); “bridging effect” for PCE with low molecular weight (top) and high molecular weight (bottom) (**b**); packing density and residual viscosity of cement paste with all 5 PCE samples as the adsorption amount increases (**c**). Authorized reprint from Ref. [62].

Generally, PCE is a type of comb-like polymer synthesized from two types of monomers (i.e., unsaturated PEG macromonomer and acrylates). The characteristic structure parameters of PCE are the molecular weight (length of backbone, *n*), side chain length (*P*), and grafting density (acid-to-ether/ester ratio, (*N* − 1)) if the chain unit distribution is statistical, while the chain unit ratio of each molecule is consistent and depends only on the feeding ratio. For a certain PCE, to compare the effects of w/b and dosage on the yield stress and the apparent viscosity of cement paste, the statistical adsorption of PCE molecules on the particle surface after adsorption can be assumed. At a high w/b ratio, the particle separation distance (surface coverage and therefore PCE dosage) required to make the paste flow is low. At this stage, the particle surface is relatively vacant for rapid adsorption of PCE. The adsorption amount will rapidly increase (almost linearly) with PCE dosage (Figure 5a,b) [52]. The adsorption ratio is high. The average separation distance will increase quickly, resulting in a lowered flocculation degree and the weakening of the particle network. The yield stress and the apparent viscosity can be reduced quickly (Figure 5b–d) [53,54]. At this stage, the viscosity of the pore solution containing a low concentration of remaining PCE is still low. However, the reduction of yield stress (1/*H*^2^) is more rapid than the apparent viscosity (only the hydrodynamic force component, local shear intensity *d*/*H* [23]). When the dosage increases to a certain value, the average separation distance is so large that particles cannot form an effective network, and bleeding occurs [32]. At this point, a lower w/b ratio is required to further increase the PCE dosage. In this phase (B), the particle surface is densely covered by PCE molecules. Still, the separation distance can be further increased with additional PCE, but its reduction effect on yield stress will not occur so readily as in the above stage (Figure 6). At this stage, the apparent viscosity gradually approaches a “plateau value” [55,56]. Further addition of PCE will make the particle surface crowded (C). The solution concentration should be raised to a high value to improve the further adsorption of PCE, due to the steric hindrance from the surface-attached PCE molecules. At this stage, the adsorption ratio is very low (generally below 30% or even 15% [55,57]). Most of the subsequent PCE addition will increase the solution concentration, indicating a rapid viscosity increase of the solution phase. However, the separation distance increase is not so obvious, and the yield stress approaches a “plateau” value [55,56], which represents the largest flow spread of the paste. As reported by Liu [57], the solution viscosity increasing at high PCE dosage is a significant contribution to the apparent viscosity increase of cement paste (Figure 5d).

Theoretically, an extremely high PCE concentration in the solution will make the particle surface fully covered by PCE molecules (condition D in Figure 5a). The lowest yield stress, denoting the upper limit of the dispersing performance at fixed w/b, should appear in this state. The “plateau” value of the flow spread after which further addition of superplasticizers will not improve the fluidity any more [55] should only depend on the adsorption layer thickness at full surface coverage. However, although experimentally we can measure and obtain a saturated adsorption amount *A*_s_ as reported elsewhere [58], kinetically this state might be difficult to reach due to the high steric hindrance [47,48] and slow adsorption rate, especially for PCE of low adsorption affinity (charge density). Therefore, the lowest yield stress in experimental testing (condition C′, Figure 5b,d) might appear at a surface coverage lower than condition D. At an even higher PCE dosage, when the PCE concentration of the interstitial solution is extremely high, the yield stress will increase due to the depletion forces [59] (Figure 5d left part). The depletion effect results from the osmotic pressure between the small inter-particle region (smaller than the characteristic size) where the polymer was depleted and the bulk polymer solution. However, if the molecular weight of PCE (M_w_ ~ 10^4^–10^5^) is relatively small, the dosage should be extremely high, which is not the common condition in application.

Additionally, the two blue filled circles in the right figure of Figure 5d indicate the lowest apparent viscosity. The PCE dosage at this point is lower than the PCE dosage required to achieve the lowest yield stress (condition C′), as demonstrated in [55,56], since the increase in solution viscosity against concentration is more sensitive than the depletion effect. When the PCE dosage is higher than the lowest apparent viscosity, the apparent viscosity increase should primarily depend on the viscosity increase of the interstitial solution. PCE with high backbone stiffness, long side chains, and high molecular weight will be more pronounced [56,62].

Generally, the capacity of reducing yield stress depends on the adsorption affinity and the adsorption conformation (*H_p_*), while the differences in the effects of PCE samples on the apparent viscosity are derived from the balance effect of adsorption layer thickness and the viscosity of the interstitial solution. If we consider that PCE is flatly adsorbed and all the side chains are flexible in the solution, the dispersing effect can be considered to only depend on the surface-attached side chains. More long side chains will induce a lower flocculation degree (higher packing density), lower yield stress, and lower apparent viscosity. As reported by [63], the dispersing performance can be determined by the adsorption amount of PCE (side chains). For PCE samples of the same side-chain length and grafting density, the packing density of the cement paste seems to be linearly correlated with the surface coverage (the side-chain density on the particle surface). The apparent viscosity is reversely correlated with the surface coverage for all the PCE samples [62].

PCE of various architectures have been investigated. Most of the former reports revealed that PCEs of higher charge density exhibit high adsorption affinity and always have a good initial plasticizing effect [64,65,66]. PCEs with long side-chain lengths are much more effective than short side-chain lengths at low w/b, due to larger particle separation distances. Thus, pastes with low apparent viscosity can be prepared with PCE with long side chains.

At a fixed theoretical acid-to-ester ratio and comparable molecular weight, by incorporation of methacrylic acid units, the PCE backbone stiffness can be elevated, resulting in improved adsorption affinity yet smaller adsorption layer thickness at full surface coverage [55]. At a high w/b ratio (0.29), PCE of moderate backbone stiffness is more effective, while the lowest yield stress (largest flow spread) at a low w/b ratio (0.22) can be achieved for PCE without methacrylic acid units. For cement paste with a high w/b, at a consistent flow spread, the apparent viscosities of pastes with these PCE samples are similar [55]. Only at a low w/b ratio, when the PCE dosage is high, will the concentration of PCE remaining in the interstitial solution be high enough to induce obvious differences. Low backbone stiffness will reduce the adsorption affinity and viscosity of the polymer solution, yet the thickness after adsorption will be high. Therefore, PCE with the highest backbone stiffness will produce paste of the highest apparent viscosity in most cases.

Adsorption affinity can also be tuned by incorporation of hydrophobic groups. The adsorption of PCE with small amount of styrene units (hydrophobic, but also increasing the backbone stiffness) can be improved, while n-butyl acrylate units reduce the adsorption affinity [56,67]. However, the hydrophobic group is not beneficial for the adsorption layer thickness; therefore, only PCE containing styrene units is slightly more effective than reference PCE at low PCE dosages. All PCEs were less effective than reference PCE at high dosages (low w/b). Additionally, PCE with styrene units reduces the apparent viscosity of cement paste at a proper low dosage, while PCE with n-butyl units increases the apparent viscosity [67].

### 3.3. Other Effects

Besides the general effects discussed above, some other mechanisms that intervene in particle interactions will also affect the yield stress of cement paste. The major mechanisms are elucidated as follows.

(1) Bridging effect: Direct connection between particles through polymer bridging or polymer-Ca(II) bridging [60,61] will enhance the particle interaction and increase the yield stress. The bridging effect is much more pronounced for large polymer molecules, especially those used for viscosity-modifying polymer admixtures [68]. Although PCE molecules are very small and the steric hindrance will prevent the interaction, a few reports have demonstrated the effect. One report compared PCEs of 4/1 and 22/1 (acid-to-side-chain ratio) in cement paste [61]. The PCE with an extremely low side-chain density (22/1) reduced the flowability of OPC paste when the dosage was over 0.004 bwoc, while the PCE of 4/1 was still effective in improving the flowability. These reports suggest that PCE bridging was the fundamental reason. Similar results were obtained in other reports and in our own experiments.

In Zhang’s report regarding the effect of the molecular weight of PCE on the rheological behavior of cement paste [62], PCE of moderate molecular weight was found to be the most effective at dispersing, although the adsorption layer thickness was evaluated to be smaller than for PCE with a high molecular weight. The underlying mechanism was assumed to be the enhanced “bridging” effect of PCE of a high molecular weight (Figure 6). Higher surface coverage was required to achieve the same yield stress as PCE with a lower molecular weight. Additionally, at a fixed flow spread, the paste comprising PCE with the highest M_w_ was the least viscous. The average particle separation distance was larger than for the PCE with a low M_w_. Although the interstitial solution viscosity was higher, the flocculation degree was lower. The local shear intensity was therefore much lower. Finally, the apparent viscosity at consistent yield stress was even lower for PCE with a high M_w_.

(2) Non-adsorbing molecules: Some reports have demonstrated that the adding of non-adsorbing PEG to the paste will increase the yield stress over a certain concentration (the critical overlapping concentration c*) [59]. The attractive interaction also creates the depletion effect. However, in the absence of superplasticizers, attractive van der Waals interaction is still the most pronounced effect that forms the interparticle network. In the presence of superplasticizers, the steric hindrance will keep particles away from each other where the attractive van der Waals interaction is weakened. The depletion force can then become more significant in enhancing the flocculation. Polymers of high molecular weight will increase the yield stress at a lower concentration.

Despite the depletion effect, some other reports have demonstrated that non-adsorbing molecules (PCE and small molecules) increase the flow spread of cement paste at a low w/b ratio [31,69,70,71]. Matsuzawa et al. [31] tested the effect of the subsequent addition of other PCE superplasticizers into a prepared paste containing a first type of PCE at full surface coverage. The results show that subsequent addition of PCE with a high grafting density lowers the adsorption but maintains the flow spread, while PCE of a moderate grafting density does not change the total adsorption but increases the flow spread. The proposed mechanism was that small molecules intercalate particles and help to prevent flocculation. Similar results have been observed for small water-soluble molecules (e.g., diethylene glycol, Jeffamine D-230) at low w/b, which can increase the flow spread at relatively low dosages (0.6 bwoc%) [69,70].

(3) Amount, size, and morphology of hydration products, which are associated with polymer structure, and exhibit pronounced effects on surface area and particle packing. The effect of early hydration products from the aluminate phase (e.g., ettringite) is more significant due to the morphology and large amount in the early stage.

A former report [72] has demonstrated that ether-type PCE (with ether-type macromonomer-containing side chains) and ester-type PCE (with ester-type macromonomer-containing side chains) will produce ettringite of different sizes. PCE with a high adsorption affinity greatly inhibits the growth of ettringite particles. However, a significant increase in the specific surface area has been observed [73]. PCE with long side chains produces ettringite with a long aspect ratio, induced by the slow growth of the lateral side of the rod-like particle [74,75].

Interestingly, the pH-value of PCE superplasticizers has been found to affect the dispersing performance [76]. Two PCE groups of different charge densities were tested. A greater difference was found between acidic and neutral PCE samples with high charge density. For PCE with a low charge density, the difference between acidic and neutral samples was not obvious. The underlying mechanism was the difference in the amount of early ettringite. In the presence of neutral PCE, more nano-ettringite formed and the yield stress of the cement paste was higher.

It is difficult to quantitatively evaluate the influence of ettringite formation on the surface coverage of PCE, since adsorption on different surfaces cannot be differentiated. However, the adsorption affinity of PCE on ettringite is stronger than for silicate phases and their hydration products. The specific surface area increase during the initial tens of minutes could be over 70% [77]. Therefore, large amounts of PCE will cover the surface of hydration products of the aluminate phase [78], which indicates the high PCE amount required to achieve a certain flowability. Additionally, theoretically rod-shaped particles (e.g., ettringite) should affect the particle packing. However, quantitative or even qualitative results regarding how ettringite particles in early hydration (especially in the presence of PCE) affect particle packing have not yet been reported.

(4) Preferential adsorption: Adsorption behavior might be different from statistical adsorption. Adsorption affinity toward particles of different types of charge and charge density should be different for the same polymer dispersant. For cement paste, the adsorption of PCE molecules on negatively charged surfaces (mineral phase, e.g., C_3_S) is much weaker than for positively charged surfaces (e.g., C_3_A, ettringite) [79]. Initially, PCE will attach to the surface of C_3_A and C_4_AF, then rapidly attach to surfaces of early hydration products, such as the alumina/ferric oxide mono/tri-sulfate phase [78,80]. By incorporating sulfonic groups, the binding capacity with Ca(II) will be reduced [81]. The adsorption on negatively charged particles is greatly reduced. Therefore, this type of PCE is always less effective than the conventional PCE with carboxylic groups. Similarly, superplasticizers will adsorb in different amounts on different particle surfaces.

(5) Multi-layer adsorption and other adsorption conformations: Multi-layer adsorption of PCE has been reported in a few papers [82,83,84]. The proposed mechanism is Ca(II) binding. However, these reports did not investigate the effect on the rheological properties at low w/b (high PCE dosage). The polymer backbone might not be flatly attached to the particle surface. Any loops will reduce the occupied surface area of a single molecule and increase the adsorption layer thickness. In Lei’s report, PCE with a high molecular weight was more effective in an alkali-activated slag system due to the high adsorption layer thickness due to loop conformation [85].

### 3.4. Effect on Thixotropy

Thixotropy of cement-based materials describes a continuous decrease in viscosity under constant shear and an increase after the reduction of the shear [86]. The change in paste viscosity is explained by the fast structural breakdown of the cement particle network during shearing and the relatively slow build-up of the microstructure at rest [27]. Previous studies [25,87] have shown that the thixotropy of cement-based materials originates from two types of structural build-up: colloidal flocculation and C-S-H bridges. Both types can be affected by PCE, leading to a change in material thixotropy.

**Figure 7:**
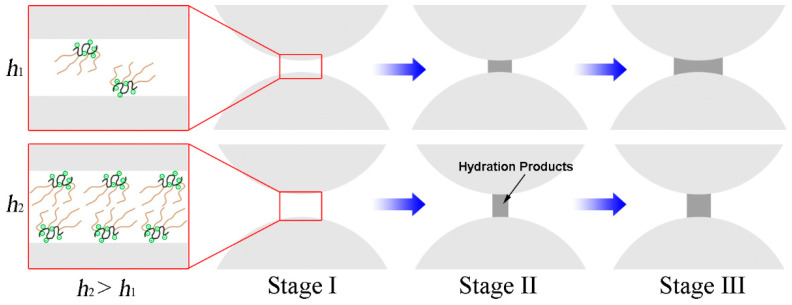
Effect of superplasticizer on thixotropic structural build-up behavior: low surface coverage (**top**) and high surface coverage (**bottom**), with equal amounts of early hydration products at different stages.

The thixotropy of cement-based materials can be reduced by PCE [86,88]. Qian et al. [86] reported that the thixotropic index, i.e., the ratio of the maximum shear stress to the equilibrium stress of cement pastes at a constant shear rate, decreases rapidly with an increasing PCE dosage and then remains approximately unchanged. The reduction in thixotropy can be attributed to the increased adsorption of PCE onto particle surfaces [58,86]. The adsorbed PCEs increase the average surface-to-surface separation distance between neighboring particles, leading to a reduction in colloidal interactions between the particles [32,63]. Thus, the build-up of the microstructure at rest due to colloidal flocculation is hindered and the thixotropy is low [58,86,88] (Figure 7). However, the low thixotropy in the presence of PCE could be related to the reduced formation of early cement hydration products. Some researchers [78,89] have suggested that the use of PCE can decrease the fast generation of nano C-S-H and ettringite. The bridging of early C-S-H between neighboring particles can be weakened. As a result, the structural build-up caused by C-S-H bridges can be slowed down, leading to lower material thixotropy.

It is known that PCEs with distinct molecular structures can cause different changes in respect of particle flocculation and early cement hydration [63,78]. Both aspects significantly affect the thixotropy of cement-based materials. However, the effect of the PCE structure on the thixotropy of the materials remains unclear. The effect of PCE side chain density and length on the thixotropic structural build-up of cement paste was reported in [90]. The results indicate that paste with PCE with a low side chain density demonstrated rapid thixotropic structural build-up, yet the tendency was not so obvious for PCE with long side chains. The thixotropy was increased for PCE with short side chains. The authors suggest that the mechanism relied on the low adsorption layer thickness and low PCE concentration in the interstitial solution, which could not slow down the build-up of the particle network due to hydration.

### 3.5. Structure Design and Performance Improvement

In application, the solution environment and powder composition of concrete always vary from each other, which affects the reducing performance of the yield stress of superplasticizers. Cements of different types and with differing contents of alkali sulfates produce pastes with varied sulfate concentrations ([SO_4_^2−^]), which greatly affect the adsorption of PCE superplasticizers through competitive adsorption. Clay can consume large amounts of PCE via intercalation. The adsorption amount of PCE for montmorillonite is ~10^1^–10^2^ times larger than for cement [91,92]. A much larger dosage is required to achieve the same flow spread. Therefore, in recent years, superplasticizers of different structures have been synthesized to achieve improvements in performance and capability for harsh situations. The structure design mainly aims to improve the adsorption of PCE molecules under different conditions or steric hindrance.

#### 3.5.1. Modification of Adsorption Behavior

An initial design to improve the adsorption on powder particles involved adding strong adsorption groups or substituting the carboxylic group with strong adsorption groups. Dicarboxylic groups (from maleic anhydride, fumaric acid), phosphate (or phosphonate) groups, or silane groups can all be introduced.

The coordination of a dicarboxylic group and a phosphate group with Ca(II) is more stable than a carboxylic group. Therefore, the adsorption affinity is improved. Dalas et al. [93] systematically investigated the adsorption of PCE with a carboxylic group, maleic acid unit, and phosphate group on calcite and synthesized ettringite in a simulated pore solution containing varied [SO_4_^2−^]. Their results demonstrate that on ettringite, which is more attractive to sulfate adsorption, PCE adsorption was more sensitive to sulfate concentration. However, under most conditions, PCE containing a maleic acid unit was less affected compared with normal PCE. Some other researchers have also tested PCE containing these groups [94,95]. Sha et al. demonstrated that PCE with fumaric acid appeared to be more effective than maleic acid and itaconic acid units both in cement suspension and a cement–silica fume system. Both the adsorbed amount and adsorption layer thickness were elevated [96,97]. Qian synthesized PCE with a maleic acid group and isopentenyl polyethylene polypropylene glycol, which improved the dispersing capacity and viscosity reduction effectiveness when the molecular weight was low and the side-chain length was short [98]. Qian suggested that small molecules with high adsorption amounts, which can also lower the surface tension, can create a water film to cover the cement particles and reduce the friction between cement particles.

**Figure 8:**
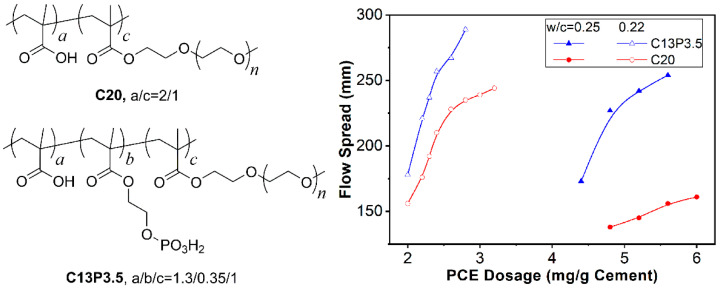
Chemical structure of different PCEs (**left**) and the performance in cement paste (**right**). Compared with C20 (containing only carboxylic groups), C13P3.5 contained a small fraction of phosphate groups. The theoretical charge density and molecular weight distribution of C13P3.5 were similar to those of C20. The performance of C13P3.5 was much better than for C20, especially at high dosages.

Many papers have investigated phosphate-containing superplasticizers [99,100,101,102,103,104,105], demonstrating superior performance against conventional PCE superplasticizers containing only carboxylic groups, derived from the high binding capacity with Ca(II). Tramaux et al. [106,107,108] reported the excellent dispersing capacity of a simulated cementitious system—aqueous dispersion of CaCO_3_ powder in a solution environment of high ionic strength. By replacing units containing a single-phosphate group with units containing double-phosphate groups, the performance could be further improved. Dalas et al. [63,93] studied the effect of phosphate groups on the sulfate sensitivity of PCE superplasticizers; phosphate-containing PCE was the most effective against sulfate competitive adsorption. Stecher et al. [109] reported the unprecedented performance of phosphate-containing PCE superplasticizers at a w/b ratio of 0.3. The performance in a V-funnel experiment indicated the prospective application in self-consolidating concrete. Stecher et al. [94] also reported the better dispersing performance, lower retarding influence, and comparable compatibility toward sulfate and clay of phosphate-containing PCE, compared with conventional PCE. We also synthesized a series of PCEs containing phosphate groups (abbreviated as P-PCE) [110,111,112]. Compared with PCE containing only carboxylic groups (C20 in Figure 8), P-PCE adsorbed more effectively on C_2_S and C_3_S. The dispersing performance of P-PCE was much better at low w/b (C13P3.5 in Figure 8), due to the lower flocculation degree and improved particle packing. Additionally, at fixed yield stress, the apparent viscosity of the cement paste was reduced by P-PCE, due to the high surface coverage of P-PCE (large average separation distance). Further, the adsorption affinity toward silica fume was improved by incorporation of phosphate groups. Thus, for the binder system of ultrahigh-performance concrete (extremely low w/b, high powder content, e.g., silica fume), the maximum flow spread of cement–silica fume paste was higher, while the apparent viscosity was lower than that with conventional PCE. Interestingly, in order to commercially lower the economic cost of P-PCE, a mixture of conventional PCE and phosphate-containing PCE also exhibited similar effects [111].

Silane groups can form covalent bonds with cement minerals, which cannot be removed from the particle surface by competitive adsorption. By the incorporation of silane groups [113,114,115,116], the adsorption on cement can be improved. PCE containing silane groups is less sensitive to sulfate. The dosage required to achieve a certain workability of concrete-equivalent mortar with cement of different sulfate alkali contents is similar for silane-group-modified PCE [116]. Results have shown that moderate substitution of carboxylic groups with silane groups is the most effective. Wang et al. reported that incorporation of silane groups can weaken the clay sensitivity of conventional PCE. Their results show that the adsorption on cement became stronger and more rapid. Although the charge density was lower, the dispersing performance in the environment was better. Intercalation was not observed.

Another design used to improve adsorption is based on conformation design. By improvement of the backbone stiffness, the adsorption affinity can be improved [51,117]. Habbaba et al. reported a new type of backbone unit that can form a cyclic structure in the backbone [117]. The synthesized PCE samples exhibited smaller conformational shrinkage under different sulfate concentrations. The dispersing performance for different types of cement was also much better than for common PCE samples.

For systems containing clay contaminants, reducing the intercalation with clay improves the performance of PCE. PCE can adsorb on the surface of clay particles and intercalate into the montmorillonite layer through hydrogen bonds [12], resulting in the exfoliation of clay particles. Generally, long PCE side chains will induce a large distance between clay particle layers (always several nanometers in a wet state). The performance of PCE with long side chains in the presence of clay particles is always not good. By synthesis of PCE using short side chains to weaken the interaction with clay [118,119,120], or incorporation of large pendent groups (e.g., beta-cyclodextrin [121,122], “snowflake”-like groups [123], or sodium alginate [124]) on the backbone, the interaction between PCE and clay can be significantly reduced. Another approach involves substituting the end hydroxy group of the PEG side chain [125,126] with a carboxylic group. When approaching the edge of the clay sheets, it is assumed that repulsion will be enhanced between negatively charged -Al-O^−^ and -Si-O^−^ and end-deprotonated carboxylic groups of PEG side chains. Intercalation will be prevented.

By regulating the rate of adsorption to gradually increase with time, the yield stress of cement paste can be kept constant or reduced against the enhancement of the particle network induced by hydration. The commercial design involves incorporating a hydrolysable backbone unit (e.g., hydroxyethyl acrylate, hydroxypropyl acrylate [127,128]). Each ester bond can be hydrolyzed under alkaline conditions to produce an additional carboxylic group for adsorption. Recently, micelle- and core–shell-structured polycarboxylates have been synthesized via emulsion polymerization [129,130]. After the gradual hydrolysis of the hydrophobic acrylate unit, disassociation of the micelle and core–shell structures occurs, releasing more carboxylic groups to promote adsorption. The slump retention performance can be extended for at least an hour. Another design involves the intercalation of polymer molecules into calcium/aluminum-type layered double hydroxides [131]. After contact with water, polymer molecules can be slowly released via ion exchange to maintain the slump of cement paste within at least 2 h.

#### 3.5.2. Topological Structure and Polymer Molecular Frame

**Figure 9:**
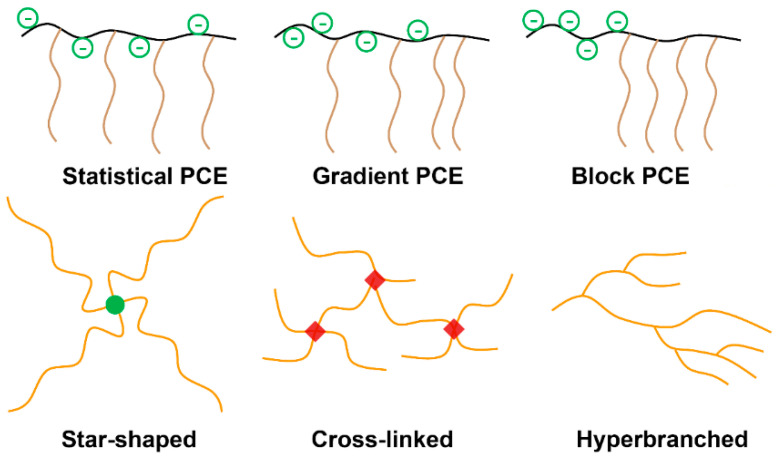
Topological structure of superplasticizers; each orange line in the bottom images represents the basic PCE structure (side chain omitted to simplify the figure).

**Figure 10:**
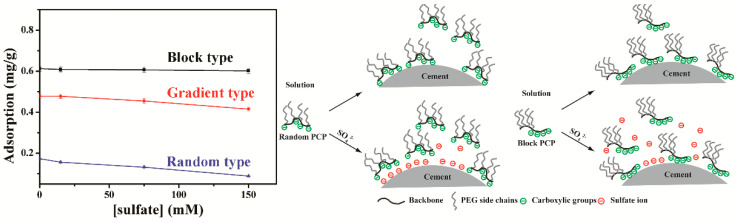
Adsorption of PCEs with different topological structures (**left**) and the working mechanism in the presence of sulfates (**right**). “Random PCP” and “Block PCP” are random-type and block-type PCE samples. Original figures from ref. [134].

The design and synthesis of PCEs of different topological structures (Figure 9) and superplasticizers of new molecular frames has enabled further development and improvements in performance. Based on a comb-like structure, gradient- and block-PCE copolymers have been synthesized via living free-radical polymerization [132,133,134,135,136,137]. The polydispersity of chain unit distribution and molecular weight can be controlled well. The adsorption groups can be concentrated in part of the polymer backbone, which is beneficial for the contact of carboxylic groups with the surface. The adsorption is more rapid and stronger than for statistical PCE. Due to concentrated side chains, the local grafting degree is also increased, indicating a potentially large adsorption layer thickness. The adsorption is much less sensitive to sulfate competitive adsorption (block < gradient < statistical) [134,135] (Figure 10). Enhanced adsorption increases the adsorption layer thickness, while a low interstitial solution concentration of PCE induces low solution viscosity. The viscosity of cement paste is generally lower [138].

So-called “star-shaped” PCEs were reported in [139,140,141]. The core-arm structures were synthesized through core units with multiple double bonds or chain transfer groups. Indeed, a core unit with multiple double bonds can act as a cross-linking site. Different results have been reported. Liu et al. reported that a star-shaped PCE with 4 arms exhibited better dispersing and slump retention performance, due to a higher adsorption amount and much stronger steric hindrance from the specified topological structure [139]. However, Li et al. reported the synthesis of a star-shaped PCE with different arm numbers in the core unit with different amounts of chain transfer groups [141]. The adsorption and initial dispersing performance was reduced with more arms grafted. The adsorption affinity was compared thermodynamically in a scaling manner. At a fixed molecular weight, the surface-occupied area became smaller for star-shaped PCEs with an increase in arm numbers, resulting in an increase in the adsorption free energy. In addition, many papers have reported cross-linked PCEs with different cross-linking monomers [142,143,144,145,146,147] with varied numbers of double bonds. Some reports have shown that slight cross-linking can improve the adsorption and thus the initial flowability of cement paste. Some other reports suggest that ester-type cross-linking monomers can improve slump retention capacity, possibly due to the hydrolysis of ester bonds. Most of the reports have indicated that a high degree of cross-linking or cross-linking with monomers containing larger amounts of double bonds are not beneficial for the dispersing performance.

Hyperbranched PCE has been synthesized via in situ oxidation-redox-initiated polymerization [148] with acrylic acid, TPEG, and DMAEMA (with an amine group as a reducing agent for initiation). The intrinsic viscosity of hyperbranched PCE was much lower than for conventional comb-like PCE, while the adsorption layer thickness was larger. The hyperbranched PCE could effectively reduce the apparent viscosity of cement paste. A jellyfish-like superplasticizer [149] was also synthesized as an effective superplasticizer. The polymers exhibited high robustness against sulfates and high flow retention capacity for cement paste.

There have also been reports in respect of modifying the molecular frame (backbone, side chain structure) of superplasticizers. As a type of biomass resource that is renewable and abundant on Earth, lignin-based materials have potential for sustainable application. Lignin-grafted polymers are superplasticizers with varied structures [150,151,152,153,154,155]. Side chains can be grafted onto the lignin frame by phenol-group-initiated radical polymerization. The grafting structure can comprise acrylic acid and a 2-acrylamido-2-tert butyl sulfonic acid copolymer, polyacrylamide, a conventional PCE structure, and polyethylene glycol chains. Reports have shown that a potentially higher steric hindrance can be ensured for lignin-grafted polymers. These materials are effective as superplasticizers. Compared with conventional PCE superplasticizers, a lignin-grafted polymer with an optimized structure might be more effective in the initial dispersion and viscosity reducing of cement paste. In the presence of large-surface-area minerals such as kaolin and clinoptilolite, lignin-grafted polyacrylamide has also been demonstrated to improve the workability of paste [155].

Researchers have synthesized polyamidoamine dendrimer-based polycarboxylic superplasticizers (PAMAM-based PC superplasticizers) [156] and polyacrylic acid copolymers with end-functionalized poly(allyl sulfonate) as side chains (PAA-g-PAS) [157]. By adjusting the generation of PAMAM dendrimers, the optimized PAMAM-based PC superplasticizers appeared to adsorb to a higher degree, and showed better water-reducing and flow-retention capacity compared with conventional PCE. The PAA-g-PAS polymer adsorbed on the cement particle surface also provided steric hindrance, while the negatively charged side chains underwent a lower degree of conformation compression in the presence of sulfates. Thus, the superplasticizer was much more effective than a conventional PCE.

In summary, polymers with an effective adsorption affinity toward cement particles and a flexible aqueous-soluble molecular frame or grafting chains to provide steric hindrance can all be considered highly effective superplasticizers. By modifying the grafted chains, the steric hindrance can be improved, compared with conventional PCEs. This enables almost unlimited chain structure flexibility toward different conditions.

### 3.6. Commercial Superplasticizer Products

Despite of the above listed state-of-the-art studies, due to economic considerations and the availability of industrial raw materials, the dominant PCE type in the market is still the conventional one, comprising so-called “ester-type PCEs” and “ether-type PCEs”. The adsorption groups generally comprise carboxylic groups and sulfonic groups. Since hydrolysis of the ester bond in a highly alkaline environment cannot be avoided, ester-type PCEs are not stable in pastes. The dispersing performance is always lower than that of ether-type PCEs.

Nowadays, in China, over 90% of commercial PCE products are ether-type PCEs, prepared via free-radical polymerization of acrylic acid, maleic anhydride, and sodium methylallyl sulfonate with different polyether macromonomers. However, small monomers are generally much more reactive in polymerization than polyether macromonomers, resulting in great differences in the yielded polymer molecules at different polymerization stages. The polymer molecule yield and chain unit composition of each polymer molecule depend on the real-time monomer concentration, which continuously changes over the whole polymerization process. Due to uncontrolled chain transfer reactions, polymer samples always have a relatively high polydispersity index.

As demonstrated by Plank et al., different polymer compositions can be obtained in the polymerization of [158] acrylic acid, α-isoprenyloxy ω-polyethyleneglycol macromonomer, and 2-trimethylammonium ethylmethacrylate chloride. The composition depends on the feeding method. Polymers with high/low acrylic acid content, cationic polymers, zwitterionic polymers, and polyacrylic acid can be produced in one batch, as indicated by charge titration. In the polymerization of acrylic acid (A) and isoprenyloxy polyethylene glycol (E), three types of triads of monomer sequences AAA, AAE, and EAE can be detected by ^13^CNMR. The composition depends on the feeding ratio. A high acrylic acid ratio will increase AAE amount, while excess acrylic acid will produce polyacrylic acid byproducts [159]. The influence of the polydispersity of PCE samples has also been investigated [160,161,162]. Although the polymer layer adsorption thickness and saturation dosage are not greatly influenced, the initial adsorption should not represent the average adsorption of the PCE samples, but should primarily be associated with large polymer molecules, which have a higher adsorption affinity than small molecules. Therefore, at low or intermediate dosages, the results depend on the polydispersity.

It should be noted that, although many papers have reported the incorporation of small functional groups or chain units within one-batch polymerization, most of them have not verified the details of the polymers. Some polymers tested were of different molecular weights. Despite the polymerization details, there are optimal values for the molecular weight and acid-to-ether ratio for each side chain length. For example, a low acid-to-ether ration will induce low adsorption, while a high acid-to-ether ratio will induce a rapid slump loss, even within the mixing process. Although a high acid-to-ether ratio will enhance the adsorption of PCE, after a short mixing time, the initial flow spread of the cement paste might be reduced. If the adsorption affinity of the PCE designed was properly adjusted compared with reference PCE (lower for reference PCE with a high acid-to-ester ratio, or higher for a low acid-to-ester ratio), improvement will be observed. However, the mechanism might be reconsidered regarding whether the performance improvement should be attributed to the structure modification or whether it is solely due to the reference PCE.

## 4. Viscosity-Modifying Admixture

Highly flowable concretes, such as self-consolidating concrete (SCC), underwater concrete, shotcrete, and so on, exhibit a high probability of segregation and bleeding of aggregates, which decrease the workability and stability of concretes [163,164,165,166]. In order to overcome these defects, a newly developed chemical admixture called a viscosity modifying admixture (VMA) has been widely used, which can effectively enhance the resistance to bleeding and segregation while improving the stability and cohesion of highly flowable concretes [167,168,169,170,171,172]. VMAs are found to exhibit thixotropic behavior, which can increase both the yield stress and plastic viscosity values of cement-based materials. VMAs can also be used as water retention or anti-washout agents to reduce the water loss in mortars and cement grouts.

### 4.1. Classification

From the chemical structure, most of the commonly used VMAs are water-soluble polymers with ultrahigh molecular weights [173], containing many hydrophilic groups. The commonly used VMAs can mainly be classified as follows.

**Figure 11:**
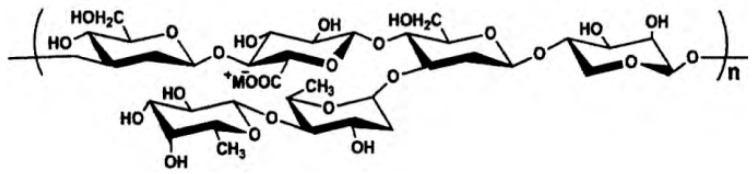
Chemical structure of diutan gum.

**Figure 12:**
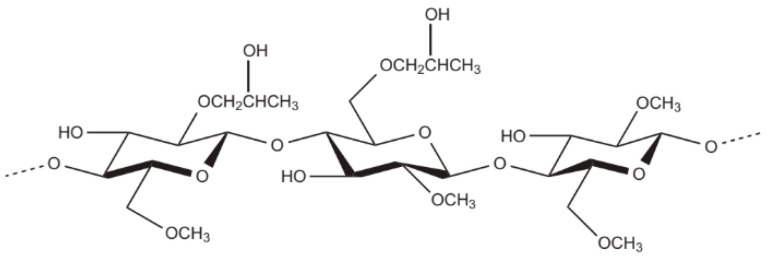
Chemical structure of hydroxypropyl methyl cellulose.

**Figure 13:**
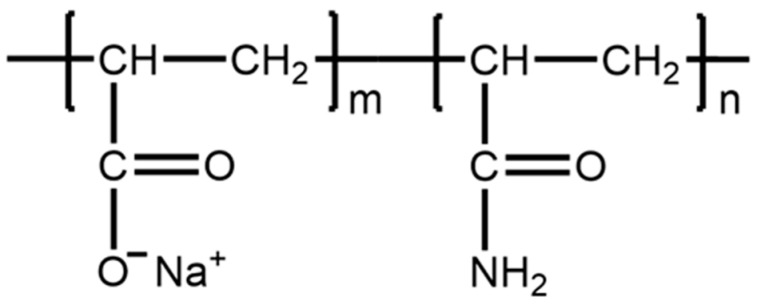
Chemical structure of poly(acrylamide-co-sodium acrylate).

(1) Natural polymers: In the early years, many natural polymers including starch, diutan gum (Figure 11), xanthan gum, welan gum, guar gum, chitosan, and plant proteins were used [174,175,176]. They are easily available from nature but usually show poor water-solubility and chemical stability.

(2) Semi-synthetic polymers: Some semi-synthetic polymers such as cellulose ether derivatives and modified starch have also been used as VMAs for concrete. Cellulose ether derivatives are the most widely used semi-synthetic polymers, including hydroxypropyl methyl cellulose (HPMC, Figure 12), hydroxyethyl cellulose (HEC), carboxymethyl cellulose (CMC), and so on [177,178,179,180,181,182,183,184,185].

(3) Synthetic polymers: In recent years, synthetic polymers, including polyacrylamide (PAM, Figure 13), polyacrylate (PAA), polyvinyl alcohol (PVA) and polyethylene oxide (PEO), have also been developed [68,186,187,188,189]. Compared with natural and semi-synthetic polymers, synthetic polymers exhibit better water-solubility and chemical stability, and can be easily chemically prepared and modified.

(4) Nanoclay: In addition to organic polymers, some inorganic nanoclays such as montmorillonite, sepiolite, magnesium aluminosilicate, and attapulgite have been used as the VMAs for concrete, and have been reported to increase the yield stress and enhance the stability of fresh cement pastes [190,191].

### 4.2. Working Mechanism

**Figure 14:**
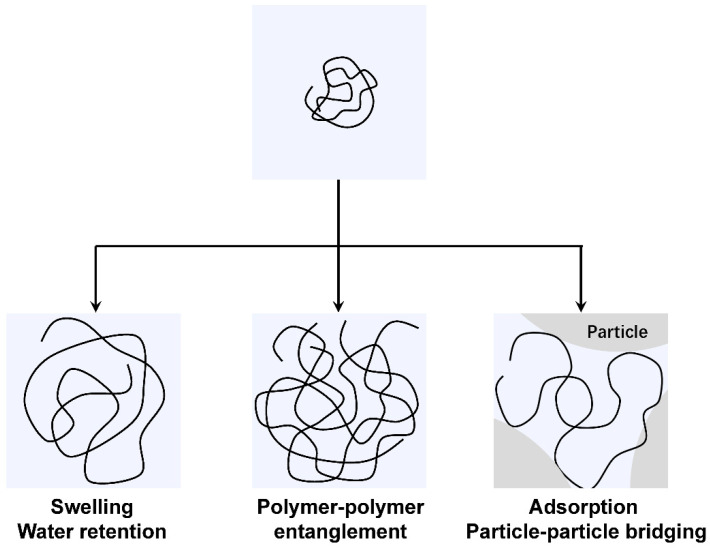
Schematic illustration of the action mechanism of VMAs.

The mechanism of VMA molecules toward cement particles is illustrated in Figure 14 and is classified as follows [192,193,194]:

(1) Water retention action: The VMA polymers contain many nonionic hydrophilic groups (such as hydroxyl and acylamine groups), which can adsorb and fix free water molecules because they have a strong ability to bind water through hydrogen bonding interactions.

(2) Polymer–polymer entanglement: The nonionic groups of the polymers have no electrostatic repulsion, meaning the polymers can easily intertwine and entangle, which results in the formation of a gel-like network. Both the water retention action and polymer–polymer entanglement increase the plastic viscosity and reduce the water loss of cement pastes.

(3) Adsorption and particle–particle bridging: The anionic groups of the polymers can adsorb on the positively charged surface of the aluminate via electrostatic attractions. In addition, the carboxyl groups can be seen as the anchoring groups for adsorption of the polymers on cement particles, and can adsorb on the negatively charged surface of the silicate via the Ca^2+^ ion bridging as a result of the complexation between the Ca^2+^ and COO^−^ [195,196,197]. The adsorption of the anionic polymers on the surface of cement particles leads to particle–particle bridging, which increases the yield stress and stability of the cement pastes. Both the polymer–polymer entanglement and particle–particle bridging result in the formation of large flocs in which free water molecules can be entrapped.

The VMA polymers can be divided into nonadsorbing and adsorbing polymers. For nonadsorbing polymers, the main action modes are water retention and polymer–polymer entanglement, which can only weakly decrease the fluidity of concrete. By contrast, the adsorbing polymers can adsorb on the surface of cement particles and lead to particle–particle bridging, which can weaken the bleeding and segregation, also enhancing the stability and workability of concrete more effectively. In addition, the adsorbing polymers can also obviously decrease the fluidity of concrete. Therefore, the increase in VMA concentration requires an increase in the superplasticizer dosage in order to maintain the targeted fluidity. In the case of a fixed dosage of superplasticizer, an increase in VMA dosage can have an adverse effect on fluidity; thus, the proper type and moderate dosage of VMAs should be selected.

Superplasticizers are polymeric dispersants used in cement-based materials. Adsorbed superplasticizers, in particular PCEs (containing long side chains), provide steric hindrance that prevents close contact between the cement particles, reducing the attractive interparticle forces [198]. Therefore, the more the PCEs adsorb, the better the cement particle dispersion. By contrast, polymers used as VMAs are quite different from PCEs. First, the molecular weights of VMAs are much higher (about 100 times) than those of PCEs. They can adsorb on the cement surface and lead to particle–particle bridging. Second, they contain no long side chains, which means there is no steric hindrance to reduce the attractive interparticle forces. Third, they contain nonionic water-binding monomers that can adsorb and fix free water molecules [199,200,201]. The adsorption of VMAs thus leads to poor cement particle dispersion.

### 4.3. Recent Advances in VMAs

The influence of VMAs on the rheological behavior of cement-based materials has been widely studied. Cellulose ether derivatives have been found to be very effective in stabilizing the rheological properties, consistency, and washout mass loss of cement pastes and fresh concretes [164,177,178,184]. The effects of welan gum, diutan gum, and guar gum on the fluidity and rheological parameters of pastes and concretes have also been discussed, showing that the increase in the dosage of the VMAs for a fixed dosage of superplasticizer increases the yield value, apparent viscosity, and plastic viscosity, and clearly reduces the fluidity [172,181,182,202]. A biotechnological biopolymer was used to prepare a biopolymer viscosity-modifying admixture (BVMA), and the rheological properties of the cement-based materials using BVMA were discussed [180]. The effects of synthetic polymers with various molecular structures on both aqueous solutions and fresh cement pastes were also fully studied [68].

The effects of different VMAs on the properties of SCCs were studied, showing that VMAs are very effective in stabilizing the rheology of SCCs [163,165,166,203]. The influence of VMAs on the compressive strength of hardened mortars and concretes was investigated, demonstrating that the effects of VMA addition on the compressive strength of mortars significantly depend on cement and superplasticizer properties [179,204,205]. The use of VMAs was validated to improve the air-void system of vibrated concretes [188], showing the stabilization effect of VMAs on entrained air bubbles in fresh concretes. The use of VMAs has also been clarified to reduce the chloride diffusion in cement-based materials [189]. The compatibility between polycarboxylate and VMAs in cement-based materials was also studied, indicating that the addition of VMAs influences the compatibility between cement and superplasticizers [9,206].

**Figure 15:**
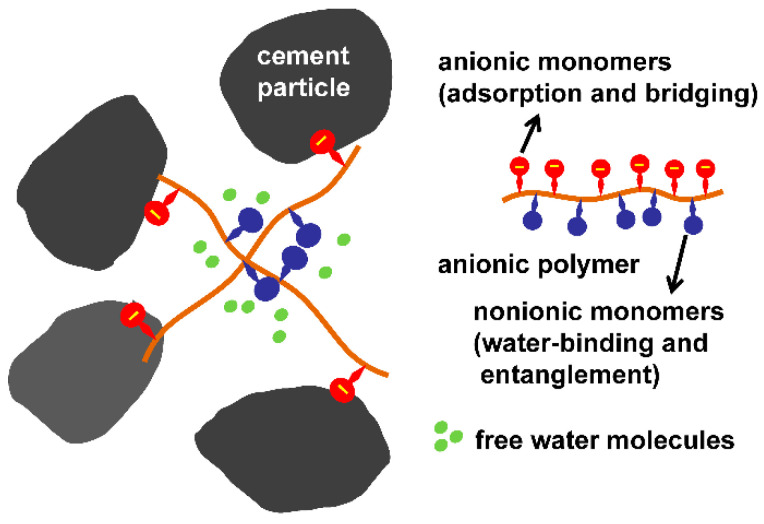
Schematic illustration of the effects of synthetic polymer VMAs on fresh cement pastes. Authorized reprint from [207].

**Figure 16:**
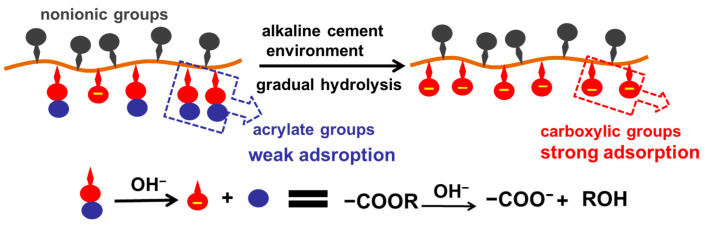
Schematic illustration of the gradual hydrolysis of acrylate-based post-acting polymers in an alkaline cement environment. Authorized reprint from [209].

The effects of the charge density, adsorption group, and molecular weight of synthetic polymer VMAs on the properties of fresh cement pastes were studied [207,208]. The adsorption, fluidity, bleeding, and rheological properties of fresh cement pastes mixed with different polymers were tested, and the structure–property relationships and action mechanisms of synthetic polymers in cement pastes were fully illustrated. The results show that the adsorption (anionic) and water-binding (nonionic) groups of the polymers affect the properties of cement pastes cooperatively (Figure 15). Acrylate-based post-acting polymers were utilized as novel VMAs for the first time [209]. The results show that the post-acting polymers were hydrolyzed and adsorbed on the surface of cement particles gradually, slowly decreasing the bleeding and stabilizing the cement pastes (Figure 16). The viscosity-modifying effects of the post-acting polymers on cement pastes were enhanced gradually and could be maintained for a long time.

## 5. Concluding Remarks

In this paper, recent advances in superplasticizers and VMAs and their effects on the rheological properties of cement-based materials have been summarized. The fundamental mechanism regarding the effect of the chemical structure of superplasticizers on the rheological properties has been revealed, based on a better understanding of conformation, adsorption thermodynamics, and the interaction with cement hydration. Superplasticizers of new topological structures and new molecular frames have been designed and synthesized. Through the adsorption or conformation adjusting, the performance of superplasticizers can therefore be improved. Additionally, more attention has been paid to the polymerization process and the polydispersity of polymer composition and molecular weight. Furthermore, the development of VMAs from natural to synthetic polymers has also been briefly reviewed. Based on the working mechanism, synthetic polymers with controllable acting process have been developed. Based on the information presented in this paper, the following conclusions can be drawn:

(1) To study the effect of organic rheology modifiers on the rheology of cement-based materials, both the physical and chemical effects of chemical admixtures on the microscale (10^−9^~10^−5^ nm) of paste should be considered. The adsorbed superplasticizer molecules provide steric hindrance against attractive colloidal forces. Part of the superplasticizer takes part in early hydration of the aluminate phase, indicating varied compatibility with cements of different types. The superplasticizer remains in the solution phase, enhancing the solution viscosity and contributing to the particle interaction. Additionally, superplasticizers affect hydration and therefore the evolution of the particle network. All these effects depend on adsorption behavior and conformation.

(2) The adsorption of PCE is driven by attractive adsorption interactions and entropy increase during adsorption. Adsorption can be improved by increasing the molecular weight, acid-to-ether/ester ratio, and backbone stiffness. Using scaling models (e.g., Flatt’s model and the surface-tethered chain model), the solution and adsorption conformation of PCE can be evaluated.

(3) Generally, with the increase in PCE dosage, at least two stages (decrease, plateau) and three stages of the evolution of yield stress and apparent viscosity, respectively, can be observed. The effect of PCE on the yield stress depends on the ALT, which is determined by the surface coverage and adsorption conformation, while the effect on apparent viscosity depends on the balance effect of ALT and the viscosity of the interstitial solution.

(4) In addition to the common condition, some other mechanisms affect the rheological behavior of cement paste. The “bridging” effect (PCE “bridged” multiple particles) enhances the yield stress. Non-adsorbing molecules can also affect the particle interaction through depletion. Preferential adsorption always occurs in a cement suspension. PCE-containing sulfonic groups are not effective compared with PCE containing only carboxylic groups, due to the low adsorption affinity toward the negatively-charged surface. PCE modifies the early hydration of the aluminate phase. The hydration products of different morphologies and surface areas form, indicating distinct particle packing and distribution of PCE molecules. Multi-layer adsorption of PCE can be observed. A “loop” conformation after adsorption will increase the ALT and improve the performance of PCE.

(5) PCE adsorption increases the particle separation distance, which reduces the colloidal flocculation at rest. Additionally, PCE reduces the formation of early cement hydration products, which reduces particle bridging. Thus, the build-up of the microstructure of cement paste is slowed down, which indicates the reduction of thixotropy.

(6) The performance of PCE can be improved through modification of the adsorption behavior, topological structure, and polymer molecular frame. Through the incorporation of functional groups of high adsorption or binding affinity (e.g., phosphate or silane groups), the performance of PCE can be improved, especially the compatibility under different conditions (e.g., against sulfate competitive adsorption or clay contaminants). Gradient and block PCEs have demonstrated enhanced adsorption against sulfate competitive adsorption. Hyperbranched PCE is effective in reducing the apparent viscosity of cement paste, due to enhanced ALT and low solution viscosity. Various types of molecular frame (e.g., PAMAM-based, lignin-based) have been synthesized and some of them have demonstrated stronger steric hindrance or improved adsorption.

(7) The difference in the polymerization reactivity of monomers results in varied chain unit composition and polydispersity of the molecular weight in the final PCE samples. The performance of PCE greatly depends on the feeding procedure. Therefore, the details of the polymers should be verified before the comparison of different PCE samples. Optimization of the polymerization procedure is also a possible way to improve the performance of PCE samples.

(8) VMAs have been widely used to enhance resistance to bleeding and segregation while improving the stability and cohesion of highly flowable concretes. The commonly used VMAs can be classified as natural polymers, semi-synthetic polymers, synthetic polymers, and nanoclays. The action mechanisms of VMA molecules toward cement particles are classified as water retention action, polymer–polymer entanglement, and adsorption and particle–particle bridging. Unlike PCEs (used as polymeric dispersants), the adsorption of VMAs usually leads to poor cement particle dispersion, which can increase both the yield stress and plastic viscosity values of cement-based materials.

## Data Availability

The data are contained within the article. Additional data are available upon request from the corresponding authors.
